# *In Silico* Modelling of Treatment-Induced Tumour Cell Kill: Developments and Advances

**DOI:** 10.1155/2012/960256

**Published:** 2012-07-12

**Authors:** Loredana G. Marcu, Wendy M. Harriss-Phillips

**Affiliations:** ^1^Department of Medical Physics, Royal Adelaide Hospital, North Terrace, Adelaide, SA 5000, Australia; ^2^School of Chemistry and Physics, University of Adelaide, Adelaide, SA 5000, Australia; ^3^Faculty of Science, University of Oradea, 410087 Oradea, Romania

## Abstract

Mathematical and stochastic computer (*in silico*) models of tumour growth and treatment response of the past and current eras are presented, outlining the aims of the models, model methodology, the key parameters used to describe the tumour system, and treatment modality applied, as well as reported outcomes from simulations. Fractionated radiotherapy, chemotherapy, and combined therapies are reviewed, providing a comprehensive overview of the modelling literature for current modellers and radiobiologists to ignite the interest of other computational scientists and health professionals of the ever evolving and clinically relevant field of tumour modelling.

## 1. The Need for *In Silico* Modelling 

Modern treatment delivery methods for external beam radiotherapy employ techniques such as intensity modulated radiation therapy (IMRT) and image guided radiation therapy (IGRT) to deliver high radiation doses to the tumour planning target volume (PTV) or multiple PTV's with ever increasing accuracy and precision. However, to further improve outcomes for individual patients it will be necessary to explore in more detail the radiobiological processes that occur in human tumours and to predict optimal treatment plans for the individual patient. There is an increasing need to incorporate cellular behaviour and characteristics into individualised treatment planning and delivery. Undoubtedly, the future of cancer research rests on a multidisciplinary approach.

The pursuit of an enhanced treatment regimen is conventionally performed through well-designed, randomised clinical trials. Clinical trials are indispensable prerequisites to establish novel therapeutic principles. Nevertheless, trials are lengthy processes which involve several influential factors for a decisive outcome: trial design, patient selection and followup, complex data analysis, and interpretation. Furthermore, trials cannot explore the sensitivity of the outcome to input parameters and covariates. 

Models are an efficient way to complement the results of clinical trials. Beside animal models and cell lines, which are often used for preclinical studies, there are computer models (*in silico* models) encompassing mathematical, physical, and engineering concepts representing the biological world. 

Models in cancer treatment are simplified tools to reproduce the biological system, thus they do not accurately reflect the fine details of the real scenario. To compensate for some of its deficiencies, the approach of computer modelling has several advantages:

- input parameters can be easily changed and results rapidly obtained;
- various mechanisms can be studied in isolation, determining their impact on specific processes;
- extreme values for different parameters may be considered, and limiting factors determined for biologically valid results;
- treatment outcome can be predicted with quantitative end points or “iso-effects”;
- models can answer the complex question of *“what if?” *

Computer models can be used to simulate tumour cell kinetics and dynamics, drug pharmacokinetics, therapies and give similar results to those in experimental tumours. Models are needed to open further research avenues and to suggest relationships between radiobiological parameters.

**Figure 1:**
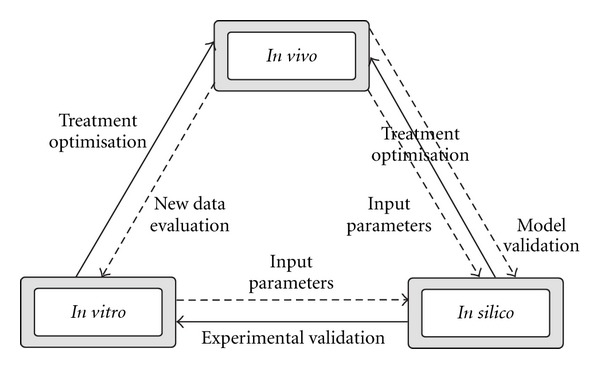
The *in silico*-*in vitro*-*in vivo* chain. The solid arrows illustrate data input whereas the dashed arrows represent the feedback data used for model validation in support of further optimisation.

The *in silico-in vitro-in vivo *chain (Figure 1) is gaining worldwide recognition among scientists and their mutual role in cancer research is illustrated through the great advances of the last decades.* In silico* models are valuable data input sources for both *in vitro *and *in vivo* models (solid arrow). Mutually, the latter offer feedback to *in silico* models in support of further developments and optimisation (dashed arrow).

When modelling the processes involved in the damage and elimination of tumour cells on the microscopic level, development of an algorithm to propagate a virtual tumour mass is first required. One approach has been to generate a full-sized macroscopic tumour mass with a predefined volume, cell type distribution, and so forth, based on average tumour statistics or a particular tumour of clinical interest; however, other modellers have used a *first principles* approach and have “grown” the virtual tumour starting from a single cell (or small group of cells). The tumour growth process is in itself a large area of research, allowing for investigations into the cell kinetics of malignant tissue. This line of study also provides modellers with ideas about how to simulate cell propagation *during treatment*, which is crucial because many treatments are delivered over multiple weeks or even months. As many of the models discussed are of the latter variety (modelling tumour growth as well as treatment), the proceeding section of this paper provides an overview of the key analytical and stochastic tumour growth models in the literature.

## 2. Tumour Growth Models 

### 2.1. Introduction

The goals of tumour modelling vary among researchers and may focus on one key biological/radiobiological mechanism or explore mechanisms and ranges of parameter values. Many models aim to identify mechanisms and parameter values at the cellular level which are responsible for macroscopic tumour outcomes, for example, the cell kinetic properties affecting the tumour growth or shrinkage rates. Modelling can add to the understanding of cellular proliferative hierarchy and the differentiation processes, evolution of genetic mutations, tumour morphology, tumour pressure gradients, angiogenesis and oxygen distributions, diffusion of nutrients, and so forth. 

The modelling methodology employed to simulate a biological system may be analytical or stochastic in nature. Analytical (mathematical) methodology is more traditional, utilising deterministic equation-based methods, often in the form of a set of equations incorporating multiple parameters. Stochastic methods use random number generation and probability distribution functions to simulate cell propagation and evolution of the tumour, for example, Monte Carlo (MC) methodology. Monte Carlo methodology enables a system to be modelled from first principles. It is a form of computation using random sampling and iteration to simulate the evolution of a physical or biological system and involves the use of probability distribution functions for decision making, for example, for the allocation of parameter values from a range of possible values. This technique is useful for modelling systems with a large number of coupled degrees of freedom, which are difficult to solve using equation-based methods.

**Figure 2:**
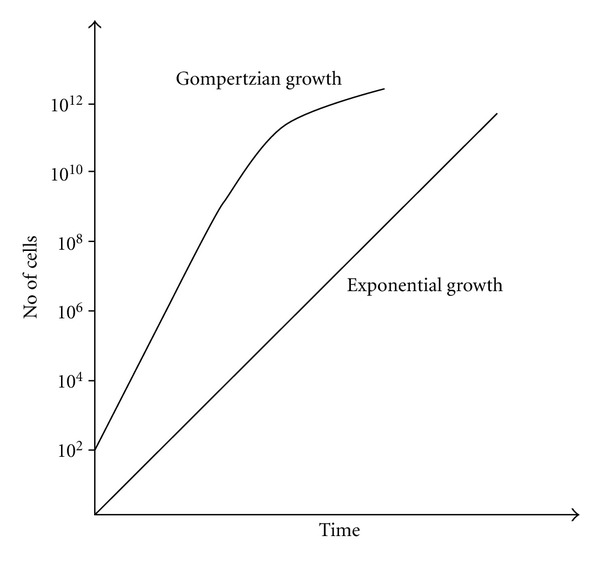
Tumour growth curves.

Tumours are complex entities, divers and heterogeneous, yet all share the ability to proliferate beyond the constraints limiting the growth in normal tissue. The growth of tumours is best represented by an exponential increase of cell number in time. Exponential growth is the simplest mode of growth assuming no cell loss or infertility. By growing exponentially, the tumour volume increases by a constant fraction in equal time intervals. Many human tumours during their growth show exponential behavior; however, there are tumours going through irregular or decelerating growth [31]. A more accurate description for the irregular tumour growth is given by the Gompertzian growth curves (Figure 2). During Gompertzian growth, the doubling time increases steadily as the tumour grows larger. The progressive slowing of Gompertzian growth may be more the result of decreased cell production rather than increased cell loss [31].

It is generally accepted that human cancers grow in an exponential or Gompertzian manner. This assumption is based on analysis of the growth of transplantable animal tumours and on averages of tumour growth in human populations. Although not valid for all individual tumours, exponential growth may accurately describe averages of human tumour growth [32]. However, there is data showing inconsistencies with exponential or Gompertzian kinetics, explainable by irregular growth kinetics. 

Model parameterisation greatly depends on the available pool of biological, biochemical, or biophysical quantities used within the equations or model algorithm. When it comes to tumour growth, modelling of both kinetic and dynamic properties are envisaged. Cellular composition, volume doubling time, growth factor, cell loss factor, labelling index, cell cycle phase-related properties (length, radiosensitivity), and oxygenation status are some of the most commonly implemented modelling parameters. Also depending on the objective of the model, treatment-related factors are often found among input parameters.

### 2.2. Avascular Tumour Growth Models

One of the earliest models of tumour growth and cell cycle simulation using the Monte Carlo approach is CELLSIM [1]. CELLSIM operates with a large initial number of cells, placed in different phases of the cell cycle whereby cells are modelled in groups rather than being followed individually. Therefore each group enters and exits a state together. When the number of groups reaches a certain limit, a reassignment algorithm will combine them making larger groups, where the new parameters are calculated using the weighted average of the previous ones. CELLSIM simulates cell cycle and distribution of cells along the cycle with and without cytotoxic treatment and it does not focus on tumour growth curves. However, the description of the model implies exponential growth behaviour.

Cellular automata models, also known as tessellation automata or cellular structure models, are one of the first types of cellular growth models developed *in silico*. These models consist of a regular grid of cells, each cell having assigned a certain state. New generations of cells are created based on predefined rules determining the state of each cell. For tumour growth simulations, cellular automata models became popular due to their ease of use. Qi et al. [33] developed such model describing the surveillance of the immune system against cancer by taking into account as main parameters the microscopic mechanisms of malignantgrowth (cell proliferation), the cytotoxic behaviours of the immune system as well as the mechanical pressure inside thetumour.The influence of these parameters on the Gompertzian growth of tumours is modelled.

Several early tumour growth models have also considered the Gompertzian distribution to describe the shape of the tumour growth curve. Gyllenberg and Webb [34] explained the Gompertzian growth curve by expressing the rates of cells transitioning reversibly between the proliferative and resting states as a function of tumour size, therefore incorporating variable growth fractions. Their mathematical model employs quiescence as a mechanism to explain characteristic Gompertz-type growth curves. The model distinguishes between two types of cells within the tumour, proliferating and quiescent. The theory behind the tumour growth model is based on empirical data suggesting that the larger the tumour, the more likely it is that a proliferating cell becomes quiescent and the more unlikely it is that a quiescent cell reenters the proliferating cycle, therefore diminishing the growth fraction. The same group [35] has later incorporated into the model a new parameter defining the size of individual cells and modelled the dependence of tumour growth on this parameter, in addition to cell transition between compartments.

A stochastic model of tumour growth and invasion looking into the relationship between the histological pattern of tumours and their functional properties was developed by Smolle and Stettner [36]. The model showed that cell division, migration, and death are influenced by both autocrine and paracrine growth factors, inducing therefore changes in tumour pattern. These findings support the observation whereby the functional properties of tumours together with the tumour microenvironment dictate the histological pattern of tumours. 

The implementation of growth regulation and control mechanisms into three-dimensional models (spatio-temporal) of epithelial cell populations was the focus of an individual cell-based model aiming to simulate the growth pattern and behaviour of a number of different epithelial cell populations from undifferentiated stem cells up to tumour cells [37]. The mechanisms implemented in the model are: cell-cell adhesion, which plays a role in the inhibition of epithelial growth at high cellular density, and cell-substrate interaction, with role in cell cycle progress. The interplay between cell parameter variation and selective knockouts of regulations and control mechanisms shows that the cell-substrate anchorage has the largest impact on the population morphology. Furthermore, the balance between the strength of cell-substrate anchorage and the trigger for contact inhibition determines the way the intrinsic cell growth time affects the population growth. The authors have underlined the advantages of individual cell-based models which are parameterized by measurable cell properties in describing the complex process of cell population growth.

### 2.3. Vascular Tumour Growth Models

When modelling clinical-sized tumours comprising up to 10^8^ to 10^10^ cells, simulating tumour oxygenation is generally considered a requirement for most tumour types in order for model growth curves to match well with *in vivo* data. Lack of oxygen in tissue, hypoxia, is commonly defined by a pO_2_ (partial pressure of oxygen) threshold of 10 mm Hg, although clinical trials may use 2.5 or 5.0 mm Hg thresholds when reporting experimental results such as the Hypoxic Fraction (HF) of cells in the tumour. It is now commonly known that low tumour oxygenation results in radioresistance and is a major contributor to treatment failure due to tumour recurrence [38]. Consequently tumour growth model research often involves the consideration of tumour cell oxygen levels and the mechanisms by which the cells receive the oxygen from blood vessels, for example, radial diffusion from cylindrical vessel to the surrounding tumour tissue. In models, the vessels may be simulated three-dimensionally within a lattice, or more simply, the final oxygen distribution to the cells may be modelled without specifically modelling the vessels themselves. 

Since the initial experimental and subsequent mathematical modelling of tissue hypoxia by Gray et al. [39] based on diffusion theory [40], interest in modelling tumour hypoxia has been relatively constant, although segmented in its aims. After initial interest in the late 1950s and into the 1970s [41–45] during which more basic mathematical diffusion based models and animal experimental work were carried out regarding tumour hypoxia and growth, the 1980s to the early 21st century saw the emergence of models that aimed to simulate vascularised tumour treatment and/or growth in the literature [2, 7, 46–51]. The modelled mechanisms of oxygen delivery and responses of the tumour in these models range from considerations of simple oxygen diffusion to considerations of oxygen consumption rates, vessel size/density/location, slowing of the cell cycle, induced cell death via necrosis and application of oxygen enhancement ratios relating to ionising radiation sensitivity. 

The 1980s saw the first real-time stochastic applications of tumour computer models, which paved the path for the complex Monte Carlo simulations of the current era. The tumour models reported on during the last decade tend to involve more detailed cell line specific kinetics and also include one or more treatment modalities with the option of individual patient data input, for example, spatial PET imaging hypoxia information, the review of which will be encompassed in the proceeding sections of this paper. However, a group concentrating on tumour growth based on the work of Anderson et al. [52, 53] provides a good example of new modelling research not only on the process of angiogenesis but also the distribution of chemical factors including VEGF, ECM, DMA, and molecular oxygen [54]. Consequently distributions of blood vessel pressure and fluid velocity related spatial distributions within a tumour could be produced and their impact on tumour growth studied. This group provides a thorough review of angiogenetic modelling in their publications. Stochastic tumour growth models from the 1980s to the present often simulate not only tumour growth dynamics but also treatment of a virtual tumour, for example, the work of Duchting et al. [7, 8], Borkenstein et al. [20, 21], Stamatakos et al. [14, 15], Harriss-Phillips et al. [29] and Marcu and Bezak [55]. 

## 3. *In Silico* Cancer Treatment Models

### 3.1. Modelling of Radiotherapy-Induced Cell Kill

Predicting the outcomes of fractionated radiotherapy using models was initially developed utilising theories such as the Power Law equation of the Nominal Standard Dose theory of fractionated cell kill [56] and various extensions to the theory, as well as multitarget and multihit cell kill models of the 1960's and 1970's, for example, Cohen's target-cell model [57]. However from the early 1980s onwards, the Linear Quadratic (LQ) model of radiation induced cell kill has dominated the literature for its use in predicting the relationship between fractionated radiation dose and cell kill for doses per fraction near 2 Gy. LQ theory was based on the pioneering work of Lea and Catcheside [58] and on the hypothesis of single strand and double strand DNA aberrations as the source of radiation induced cell damage. 

Use of the standard LQ equation became extremely popular in the mid 1980s and was soon extended by many authors by the addition of various modification factors for modelling effects such as the “time-factor” [59] for rapidly responding tumours and the oxygenation enhancement ratio (OER) [60] for tumours experiencing radioresistance due to hypoxia. Other equation-based models such as the binary misrepair model and models of repair capacity saturation were also devised and later compared to the LQ model [61], which yielded similar results under specific conditions, for example, 2 Gy per fraction. Dale and Jones have provided a thorough review of the history of mathematical fractionated radiotherapy models [62], which is recommended as a key text for all tumour model researchers, as are review articles by Fowler [63, 64] and Bentzen [65].

Alongside the development and widespread clinical implementation of the LQ model, other models were emerging in the last two decades of the 20th century. These models were initially mathematical based; however, a subset of modellers soon branched out into the utilisation of stochastic modelling techniques to describe the action and impact of ionising radiation on living tissue. As the models became more sophisticated, so too did the requirement not only to describe the effects of radiation more accurately, but also to model on smaller and smaller scales. Imaging modalities were beginning to collect data on the “mm” to cellular scales depending on the modality used, creating huge data sets when modelling tumours of macroscopic volumes.

Macroscopic tumour modelling is most relevant for comparisons to human data and eventual translation into clinical use, consequently the modelling of the tumour vascular system became of interest. Inclusion of oxygenation parameters in models was driven by the increasing realisation of the importance of tumour hypoxia as reported from large clinical trials for certain tumour sites, for example, head and neck [66–68]. Consequently, use of techniques and theories such as diffusion theory for the transport of oxygen through tissue [40] were employed by modellers. This particular theory was not a new one; however, theories such as this were now becoming increasingly utilised and incorporated into new sophisticated mathematical and hybrid mathematical-stochastic (automaton) models [6, 45]. 

Due to the advances and increasing usability of personal digital computers in the 1980s, the field of tumour and treatment modelling was boosted, particularly in the US and in European countries such as Germany and Norway. One of the first stochastic tumour growth and/or radiotherapy models of avascular tumours was called *CELLSIM* and later the 2D *CELLGROW *[1, 2], as previously mentioned in this paper. The treatment component of this model involves randomised “cell groups” (representing groups of cells) death based on LQ surviving fractions (SF) and focuses on cell cycle kinetics the impact of cell grouping on the statistical outcomes of phase blocking and cytotoxic drugs.

In the following decade, a mathematical model describing the changes in tumour growth during development and LQ-based cell kill emerged [3], simulating exponential cell proliferation at small sizes and Gompertzian-shaped growth at larger sizes. Deterministic equations are used to plot tumour mass curves as a function of time. Radiotherapy is modelled, with varying regimes, and the surviving cell fraction determined as a function of time. In the 1980s and well into the 1990s, groups lead by Duchting and Kocher were each developing stochastic models of tumour growth and radiotherapy. Duchting et al's. work was the first to explore cell by cell modelling of a tumour cell population in a growth medium (tumour spheroids), with the inclusion of cellular oxygenation parameters as well as many cellular kinetic and radiation schedule parameters (six schedules reported on) available for modification by the user [7–10]. Kocher et al.'s work [11, 12] also considered cellular oxygenation by modelling a regular array of vessels in 3D within a tumour mass, with 3 different schedules simulated. Both groups modelled slow versus rapid growth kinetic and studied the impact on treatment response, and either manipulated with the LQ equation by means of single OER values or unique *α* and *β* parameter values for hypoxic tissue.

Another mathematical model was reported in the late 1990s from Wouters and group [13, 69], which was also concerned with modelling tumour oxygenation and also the process of reoxygenation, considering two levels of oxic status versus a full range of pO_2_ values. In the next few years a stochastic model of tumour growth was developed, simulating cells with ranges of proliferative capacity (epithelial hierarchy), and investigating the kinetics of accelerated repopulation and treatments including radiotherapy and chemotherapy [4, 5, 70]. The model showed that while cellular recruitment from the quiescent phase into the cell cycle does not constitute a key mechanism in tumour repopulation after radiotherapy, loss of asymmetry in stem cell division, even for a small percent of stem cells, could be the key process in tumour regrowth.

In 2002, two mathematical tumour treatment models incorporating stochastic parameter distribution were reported on, one aiming to model and clinically verify accurate microvascular density and heterogeneity in a 2D tumour cross section with cylindrical vessels [18, 19] and the other simulating the delivering altered doses to different cells based on oxygen status. In the later model, chronic (permanent) as well as acute (temporal) hypoxia was investigated, and overall a 20% to 50% boost in dose to the hypoxic cell population (up to 20% chronic hypoxic volume) required the same dose to control the tumour as for an oxic tumour.

Søvik and colleagues [27] have developed a model aiming to “dose-paint” radioresistant tumour subvolumes with higher than normal doses, using clinically relevant oxygenation distributions in a mathematical spatial automaton model. Reoxygenation was also considered. The group concluded that prescribing varying doses to different parts of the tumour can significantly increase tumour control probability although the rate of reoxygenation was found to be the crucial parameter. Tumours with no reoxygenation had the most benefit of dose redistribution. The level of chronic hypoxia influenced outcome more than the level of acute hypoxia. 

The modelling work of Daşu et al. [23, 71, 72] explored not only the measurement process of key parameters in tumour models and the effects of hypofractionation, but also the effects of chronic and acute hypoxia on tumour control in a mathematical probabilistic model [24, 25]. Results justified the need for a full description of tumour oxygenation to predict treatment outcomes and showed that temporal oxygenation changes between treatment fractions are less important than the presence of chronic hypoxia.

Finally, there have been a number of stochastic vascularised tumour growth and/or radiotherapy treatment models produced in the past decade. Of those reviewed herein, one is a purely temporal model, while the others are spatial-temporal. In general, most modern models simulate cell growth on an individual cell basis; however, there is a trend in some models to average cellular properties and model “tumour voxels” or “geometrical” cells. This is often performed because of the large tumour volumes being applied and owing to the direct input of imaging (anatomy and/or functional) data into the models. This direct input is used in some model as an alternative to simulating the tumour growth process, and rather the tumour is created at full size based on clinical data, ready for treatment simulation. There are advantages and disadvantages to this technique, including the lack of understanding/research into microscopic aspects of the tumour, that is, cellular kinetics and how kinetic parameters change with tumour volume, but also the benefits of individualising the model for a particular patient and therefore providing more direct outcomes to compare with clinical data. 

The Greek modelling group led by Stamatakos have published many papers regarding their models of growth and radiotherapy for lung and brain tumours. Initial work began with a 3D discrete time step model [16] (extending upon pioneering work by Duechting in the 1980s) with spatial visualisation and modelling *in vitro *tumour spheroids for small lung cell carcinoma. Since 2004, the group has published models of *in vivo *tumour systems [14, 15, 17, 73–75], concentrating on glioblastoma multiform and the incorporation of experimental and clinical data into the model. The model now uses the concept of a “geometrical cell” (GC) to average cellular properties obtained from imaging data and uses a grid size of up to 120^3^. A comparison between six radiotherapy fractionation schedules has been performed by varying parameters such as the cell loss factor, OER, OER_*β*_, cell cycle time and mutated versus wild type p53 status. Results show that accelerated schedules are superior to conventionally fractionated ones and that wild type tumours (higher **α*/*β**) respond well compared to mutated tumours.

Borkenstein et al. [20] considered angiogenesis in their work and used an individual cell approach to spatially model tumour growth and radiotherapy treatment. Capillaries are placed at intervals in a 3D lattice, with cellular oxygenation based on the distance to the nearest capillary cell. Cells in a hypoxic state secrete an angiogenesis factor in proportion to the number of hypoxic cells in the tumour. Radiotherapy is based on LQ theory, with reoxygenation and accelerated repopulation also modelled. Various radiotherapy schedules have been compared, using OER values of 3.0 and 2.5. Results show that total doses of 86 Gy versus 78 Gy are required to achieve tumour control for conventional and accelerated schedules, respectively. Harting has extended the work by modelling a hypoxia-induced angiogenesis factor excreted radially from hypoxic cells [21, 22].

*HYP-RT *is a temporal stochastic model [29, 30], simulating individual tumour cell division and the effects of fractionated radiotherapy, with assumed randomised spatial cell placement in the tumour. The model is based on the proliferative hierarchy of epithelial cells, simulating head and neck squamous cell carcinoma growth and radiotherapy, with hypoxia modelled using realistic oxygen distributions and a dose per fraction dependent OER curve. The model is capable of simulating the effects of reoxygenation of hypoxic tumours as well as accelerated repopulation. Results show that accelerated repopulation and the percentage of stem cells are the two most important parameters controlling growth rate and radiotherapy outcome. An average simulated hypoxic tumour requires an extra 16 Gy in total dose to achieve tumour control using conventional fractionation. Accelerated repopulation had the effects of requiring an increase in dose per fraction of 0.5 to 1.0 Gy to control the extra cell growth. Hyperfractionated schedules using 2 × 1.1 Gy per day were found to be most effective, justified by efficient cell kill and relatively low early and late normal tissue toxicities, as predicted by biological effective dose. 

A stochastic tumour simulation model [28] using cell line specific parameters and functional pretreatment PET/CT data was developed to investigate the effects of oxygenation on the radiation therapy outcome for HNSCC. Rather than using a three-dimensional lattice, this group uses a one-dimensional list of “cell groups” to store temporal cellular data, in order to minimise the number of stochastic calculations and processes.Patient data is imported (oxygenation and proliferation information) based on image voxels from PET scans, with one voxel representing approximately 10^6^ cells. LQ cell kill is applied to simulate radiotherapy, with OER values for voxels taken into account. Chronic hypoxia and reoxygenation is considered, as were individual cell cycle phases and radiosensitivities. The results show that tumour responses vary as tumour oxygenation levels decrease and that oxygenation varied in time throughout treatment in a similar manner to human tumours. Tissue growth curves followed *in vitro *cell line data for un-irradiated and irradiated cell lines (*HNSCC-1*), with an accurate time delay of tumour shrinkage predicted.

As mentioned in a number of the preceding model outlines, the majority of modern models use the standard LQ theory as a basis for stochastic cell kill; however, some models attempt to readdress cell survival theory by means of new equation sets. Such an example of this is the model by Hanin and Zaider [76] who have aimed to develop a cell survival model accounting for micro-dosimetric effects of radiation damage. Poisson based theory of DNA damage is no longer followed, instead normal shaped distributions are utilised making the model potentially suited to low, intermediate and high dose per fraction regimens.

*In-silico* tumour growth and/or treatment models have various degrees of complexity, with numerous assumptions applied, as is inherent in all modelling applications. Commonly, the goal of the modeller is to determine the optimal treatment strategy, for example, dose fraction sizes and timing, to achieve tumour control or total cell kill for a specific tumour type. The goal should also be to achieve optimised tumour cell kill within minimal normal tissue toxicity levels, ideally on an individual patient basis. This goal, however, is very challenging for reasons including: data gathering from reliable *in vivo* experiments, the large and varied patient sets in clinical trials and the extremely complex biological and chemical processes involved in carcinogenesis and tumour evolution. Tumour radiotherapy modelling requires not only accurate radiation damage models but also the implementation of how the tumour cells respond to sublethal or lethal damage, which may change with dose received, tumour volume, oxygenation levels and numerous other cell line specific and individual tumour-based factors. Nevertheless, the development of models has and will continue to assist radiobiologists and clinicians in predicting tumour behaviour and understanding microscopic mechanisms and impact upon macroscopic and measurable tumour parameters, and as such the research should continue and be encouraged.

A selection of key tumour models from the literature are tabulated below regarding avascular tumour radiotherapy models (Table 1) and vascular tumours (Table 2). Readers should note that many hundreds if not thousands of articles have been published in peer-reviewed scientific journals on expansions and clinical data fitting to the models mentioned in this paper and many other models in the literature (for example, the use of mathematical distributions of values for radiosensitivity parameters or oxygenation parameters, as well as splitting up the equations into those tailored specifically for late and early responding tissues [77–81]). However, it is beyond the scope of the current review to explore the use and/or interpretation of clinical data by means of *in silico* radiotherapy models.

While the vast majority of tumour growth and treatment response models (such as the ones presented above) are simulated on a cellular level, models on a smaller scale are a hot topic of current investigation. Monte Carlo models targeting the cell nucleus and cytoplasm have been developed using the ever-expending GEANT4 MC simulation toolkit. Barberet et al. have modelled different cell geometries found in a typical cell population in order to evaluate the absorbed energy from alpha particles and their response to different irradiation protocols [82]. The pictorial results of this subcellular model of energy deposition after alpha-particle interaction were in agreement with the experimentally obtained images of DNA double strand breaks signalling proteins. Absorbed dose after cellular irradiation on a nano-scale was the focus of another, technically similar paper, incorporating different alpha-particle sources [83]. While the emphasis of these papers is on the microdosimetric aspects of radiation interaction with tumour cells, this different perspective can bring more insight into the radiobiological processes on a nano-scale, therefore a better understanding of the impact of radiation on cell behaviour in its microenvironment.

### 3.2. Modelling of Chemotherapy-Induced Cell Kill

Chemotherapy agents act via many different pathways and they vastly differ in biochemical structure, molecular mode of action, pharmacology, clearance, and side-effects. Furthermore, in chemotherapy there is no formalism equivalent to the linear quadratic model used in radiotherapy which would describe, in a simplistic yet practical way, cell survival. 

While quantitative modelling has great potential, it requires knowledge of the numerical values of multiple parameters in order to characterize the chemotherapy dose regimens. This information is rarely available; therefore, the modelling of combination-chemotherapy regimens (drug cocktails), or of large classes of chemotherapy agents, can induce errors and inaccuracies in the simulation process.

The pool of chemotherapy-induced cell kill models is vast. Similarly to radiotherapy, chemotherapy models are divided between analytical and stochastic and they target various aspects of drug kinetics/dynamics and tumour response. In the paragraphs below, the main chemotherapy model categories are presented by listing the most representative papers from each group. Besides the most common cell kill models that investigate the correlation between tumour kill and drug concentrations/exposure times (area under the time-concentration curve), there are models studying the effect of various drugs on tumour cell kill along the cell cycle as well as compartment models focusing on drug pharmacokinetics. In addition, there are models which tackle the problem of drug resistance and repopulation during chemotherapy—two major factors which can lead to treatment failure. While models of drug resistance are more commonly reported in the literature, tumour repopulation during chemotherapy is usually a neglected factor when it comes to modelling.

One of the first reviews on chemotherapy modelling was published by Aroesty et al. [84]. The main focus of the paper was on the effects of cell-cycle specific therapy on tumour growth and on the distribution of cells along the cell cycle. Several mathematical models of chemotherapy were developed afterwards considering generalized analytical models for cycle-specific and cycle-non-specific therapies, respectively. The main differences between these two categories lie in the parameters quantifying cycle specificity and variation in growth fraction [85]. Many such models assume that the growth fraction of the tumour cell population responds instantaneously to cell killing by chemotherapy. This is not realistic, as drug pharmacokinetics indicates the existence of a drug “binding time” to achieve cell damage, which does not occur instantaneously. Some chemotherapeutic agents need several hours to form cytotoxic DNA adducts [86]. Moreover, numerous drugs express cytostatic properties, thus arresting the cells into one of the cycle phases before dying. Cell arrest can last for days [87]; therefore, the instantaneous kill is not validated.

Despite the obstacles imposed by chemotherapy modelling, models of drug pharmacokinetics and pharmacodynamics have been developed in the past, using either analytical or probabilistic methods [85]. In fact, the first model to describe pharmacodynamic effects by means of drug-induced tumour growth inhibition *in vitro *was reported by Hill in 1910 [88]. Hill's model, also called the logistic model, is still commonly used to illustrate the concentration-effect relationship for various drugs based on statistical fits to a sigmoidal curve. However, when cell cycle specificity comes into play, the model has its own limitations. 

There are several models studying the effect of various drug concentrations and exposure times on tumour control. Gardner [89] proposed an exponential kill model to predict the shape of dose-response curves based on several parameters: cycle phase specificity of the drug, cycle time, drug concentration, and exposure time. The analytical equations presented are able to predict the inhibitory concentration to achieve a certain percentage of cell kill.

Numerous studies have used the “area under the time-concentration curve” (AUC) as an approach to model chemotherapy. The area under the curve is a commonly used measure of total drug exposure and is obtained by plotting the concentration of the agent as a function of time and obtaining AUC by integration. While for some drugs (like alkylating agents) the effect is proportional to the AUC [90], for others, the duration of exposure may be more important than concentration; therefore, the relationship between AUC and tumour response is weaker. For certain drugs, such as Cisplatin, studies show that AUC is a good predictor of response [91–93]. Moreover, since the magnitude of exposure to Cisplatin is, through the DNA-adducts formation, the major determinant of the response rate, the AUA (area under the DNA adduct-time curve), also offers a reliable prediction in tumour response [93]. 

The AUC models are usually based on *in vitro* data regarding time dependency of drug potency, slope of the concentration-effect curves, and relative degree of drug resistance. Levasseur et al. [92] have created a pharmacodynamic model to facilitate the quantitative assessment of the growth-inhibitory effect of anticancer agents as a function of concentration and exposure time. Empirical mathematical expressions were built into a global concentration-time-effect model which showed that it was possible to modulate drug effect, response heterogeneity, and drug resistance by altering the time of exposure to the agents.

**Compartment Models**

**Figure 3:**
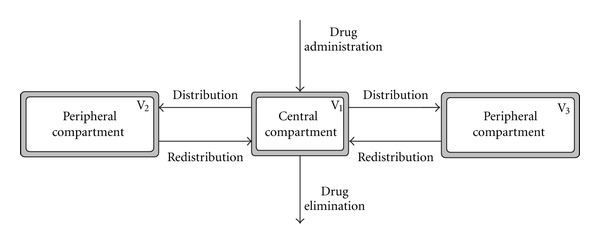
Schematic representation of a three-compartment model.

There are convenient ways to describe drug pharmacokinetics inside the body, that is, the way plasma concentration of a drug changes over time. Depending on their properties (distribution, metabolism, clearance) drugs follow multicompartmental behaviour (usually two or three-compartment models) (Figure 3). The common aim of these models is to find optimal tumour control in cancer chemotherapy via cell cycle specificity. One-compartment models are very simplistic as they consider the whole body as a single unit (compartment) in which the drug concentration is assumed to be uniform. This assumption is not valid for tumours, as the uptake of chemotherapeutic agents varies as a function of cellular proliferation (cytotoxic agents preponderantly target cells with high mitotic index). In two compartment models drug disposition is biexponential, whereby the drug is distributed into a second compartment but is eliminated from the first. Three compartment models are more complex as they have two peripheral compartments where drugs are distributed before elimination from the central compartment. Compartment models are commonly used in PET studies to evaluate the pharmacokinetics of specific radioisotopes.

On a cellular level, compartment models are designed for investigations into cell-cycle kinetics. In these models, the compartments are represented by the phases of the cell cycle, starting with the growth phase G_1_ which leads into the DNA synthesis phase S, followed by another growth phase G_2_ which then leads to mitosis, M. The options are to divide the cell cycle into two (usually G_1_ + S and G_2_ + M) or three compartments (usually G_1_, S and G_2_ + M). This division into compartments facilitates the modelling of cell cycle-specific drugs, that is, different classes of chemotherapeutic agents (such as cytotoxic agents, cytostatic agents or recruiting agents—when the G_0_ resting phase is also taken into account). This type of cell cycle kinetics modelling was introduced by Swierniak et al. in the 1980s [94] and later extended into more complex simulations [95, 96]. 

Chemo-related factors such as drug diffusion, uptake/binding, clearance and their effect on cell cycle progression are individually incorporated in various spatio-temporal models [97–99]. A more complex mathematical simulation has accounted for all the above parameters with the intention of modelling the interaction between drugs and the heterogeneous tumour microenvironment [100]. In this work, a multicompartment pharmacokinetic analysis of two drugs (paclitaxel and 5-fluorouracil) with different transport characteristics is modelled with emphasis on drug diffusion, clearance, and cytotoxicity, leading to a cell distribution along the cycle similar to experimentally obtained data. A major finding of the simulation was the observation that cell-cycle specific drugs might not offer unique advantages over cell-cycle nonspecific drugs. An added result of the model is the chemoresponsiveness to paclitaxel of slowly growing tumours which was shown to be higher than of fast growing ones due to high repopulation in between cycles of chemotherapy in the latter tumour group (which could not be compensated by the drug's cytotoxic effect). 

Another multi-compartment model to predict cellular response of different cell lines to mitotic arrest when exposed to paclitaxel was developed by Basse et al. [101] with statistical results validated against flow cytometry analyses. 

On similar principles of compartment modelling, the pharmacodynamics of Cisplatin have been simulated [91] by considering transport reaction processes between extracellular and intracellular compartments, with drug species classified into extracellular concentration, intracellular concentration, concentration bound to DNA and concentration released from DNA as a result of DNA repair. The model is based on the assumption that cell kill depends on the peak level of DNA-bound intracellular platinum and for short exposure times it yields predictions similar to those resulting from AUC-type models. The major problems with compartment models, as with any other chemotherapy simulations, are the identification of cell cycle parameters which can influence drug kinetics, thus short- and long-term effects.

One of the major challenges of chemotherapy is associated with drug resistance caused by mutations in cancer cells. *Models of drug resistance* started to be developed in the late 1970s, with the Goldie Coldman [102] model on the theory of evolution of drug resistance by clonal selection. Their model was based on biological assumptions stating that drug resistance results from clonal selection of randomly occurring mutants which are completely impervious to the drug. The analytical model followed the development of the mutant cell population as well as the sensitive tumour cells, considering that the same growth kinetics applies to both groups of cells. Clinically, such an assumption is not realistic, as sensitive cells are killed more easily than mutant resistant ones. Another oversimplification of their model was to consider drug resistance as all-or-none (mutants, deemed to be more resistant, were completely unreceptive to the drug). Therefore, progressively higher levels of resistance can be expected to emerge with continued treatment. 

Birkhead et al. [103] have designed a model that brings the principles of the Goldie-Coldman model closer to the actual clinical practice. The modelled tumour includes three cell categories: cells presenting with intrinsic resistance, the second group characterized by acquired resistance, and the third group being a sensitive population, responsive to the drug. Various drug concentrations are administered to study tumour response in time. The model relates to chemotherapy treatment in general, therefore, in order to simulate treatment strategies, specific values have to be used for cell-kill and resistance. This requirement is a limitation to the model, due to the lack of biological data and the uncertainties in the existing values for larger groups of patients.

Mathematical models of gene amplification were developed by Kimmel and Axelrod [104] to study cellular drug resistance. Their models are based on the principle whereby, at each cell generation, there is a probability of increasing/decreasing the gene copies per cell. The consequence of this probabilistic mechanism is reflected in the increased number of genes (gene amplification) found in tumour cells which confer resistance to chemotherapeutic agents. The novelty of the model lies in the description of the probabilities of the changes in numbers of gene copies in each cell, that is, the rates of gene amplification and deamplification. The model investigates the mechanisms and conditions responsible for a stable distribution of the number of gene copies as supported by experimental data.

Ample modeling of drug resistance was undertaken by Komarova [105] and Komarova and Wodarz [106]. Using a stochastic approach involving a discrete state space Markov process, their model allocates each cell type resistance properties. Cells can acquire resistance to drugs by means of mutations (where resistance to one drug does not imply resistance to another drug). In order to develop resistance to a number of *n* drugs, a cell must accumulate *n* mutations. Drug-induced cell death is regulated by the extent of drug resistance, whereby cells resistant to all drugs are not killed by the drug while cells susceptible to certain drugs are labelled with a drug-induced death rate dependent on treatment intensity. The model concluded that the success of the treatment is independent on turnover rate (the ratio of the natural death rate and replication rate) for one-drug treatments but dependent on turnover rate for multiple-drug treatments. A more specific Monte Carlo model, looking into tumour resistance to Cisplatin was developed by Marcu et al. [107] by modelling two classes of drug-resistance mechanisms: one leading to low drug uptake and the other responsible for the decreased susceptibility to the induction of apoptosis. To quantify the extent of drug resistance, the Cisplatin resistance factor (CRF) was defined. Drug resistance was shown to be a cumulative process: for low drug uptake, resistance seemed to cumulate linearly or even supralinearly for very low uptake. When decreased susceptibility to the induction of apoptosis was modelled, resistance increased over a sigmoid pattern.

Modelling of crossresistance in cyclic chemotherapy treatment was tackled by Katouli and Komarova [108] showing that the general rule in cyclic treatment in order to avoid cross-resistance would be “best-drug-first, worst-drug-longer,” meaning that the optimal strategy is to start the chemocycle with the more powerful drug but use longer cycles for the weaker drug.

While repopulation during radiotherapy is an extensively studied aspect associated with treatment failure, *tumour repopulation during chemotherapy *is usually a neglected factor. As shown by Davis and Tannock [109] the impact of repopulation between cycles of chemotherapy on treatment response may be comparable to that of intrinsic or acquired chemoresistance. These findings have been confirmed by a stochastic model of tumour growth and response to chemotherapy [110]. Cellular recruitment was modelled by releasing various percentages of quiescent cells into the mitotic cycle after each drug-caused cell kill. The onset of repopulation was also simulated, with both immediate onset and late onset of cell recruitment. Repopulation during chemotherapy was shown to be a highly potent phenomenon; similar to drug resistance, therefore it should not be neglected during treatment. 

Models are useful tools to simulate novel treatment schedules designed to improve treatment outcome by means of higher therapeutic ratio. Such a novel regimen for Cisplatin was suggested by Marcu and Bezak [55] in a neoadjuvant setting, based on previously grown tumour model using probabilistic methods of tumour development and response to therapy. The proposed model suggests that Cisplatin be delivered every three days leads to similar tumour control as the daily regimen, but with better organ sparing and higher therapeutic ratio than the weekly drug schedule. 

Mechanistic mathematical models developed to improve the design of chemotherapy regimen were summarised by Gardner [111] and Gardner and Fernandes [112]. Mechanistic models are tools which incorporate patient-specific cell kinetic parameters and allow for prediction of heterogeneous outcome across patients. Such models, based on drug pharmacokinetics and dynamics incorporating subpopulation drug resistance, cell division, and apoptotic rates were designed by Gardner [113] to kinetically tailor treatment (KITT model) to individual patients. An impressive number of 26896 tumours were modelled to build a decision tree for prognosis and the simulated predictions were in good accord with clinical trial results. These models are needed to explain multiple-drug interactions, the evolution of drug resistance inside tumour cells, cellular kinetics and the choice of chemotherapeutical agents. 

The literature on chemomodelling shows that optimum treatment strategies are hard to derive mainly because of lack of quantitative knowledge of the biological parameters of cancer chemotherapy. While quantitative data for a specific drug can be obtained from specific *in vitro* experiments, the number of unknown parameters originating from multidrug interaction turns modelling into an exigent task. 

### 3.3. Modelling of Tumour Response to Combined Treatment

The literature is scarce on combined chemoradiotherapy models, a possible reason being the lack of quantitative experimental data on radiation-drug interactions. Studies which model combined treatment techniques aim their focus towards factors such as (1) timing between agents, (2) optimal combined schedules, (3) the extent of agent interaction (additive, antagonistic, or synergistic), (4) dose-effect and (5) acquired resistance to treatment, to name just a few. 

Goldie and coworkers [114] simulated alternating chemotherapy and radiation on a hepatoma, based on experimental data. The model was built on a previously developed tumour growth model with three discrete compartments: stem cells, differentiating cells, and end cells. The main focus of the model was on stem cells, which have been classified into various resistant groups: cells resistant to chemotherapy but radiation sensitive, cells resistant to radiation but chemo sensitive, cells sensitive to both treatments, with the final group of cells resistant to both therapies. The aim of the combined model was to alternate radiotherapy with chemotherapy (cyclophosphamide) in various protocols in order to achieve an optimal tumour control. They have concluded that combined regimen is more effective in eliminating the stem cells than any of the two modality regimens alone. 

Cisplatin and radiotherapy for advanced head and neck cancers was stochastically modelled by Marcu et al. [70] looking at cell survival (tumour control) after various combined schedules. The treatment modules were applied on a previously developed virtual tumour consisting of kinetic parameters characteristic to squamous cell carcinomas of the head and neck. The model showed that while weekly Cisplatin, which is the current standard of care, has improved radiotherapy by only 6%, daily administration of Cisplatin led to a 35% improvement in tumour control as compared to radiation alone. Furthermore, optimal treatment outcome was obtained when Cisplatin was administered very closely to radiotherapy (immediately before or after irradiation) due to Cisplatin pharmacokinetics. 

Besides radiotherapy and chemotherapy, immunotherapy has become an important aspect of today's cancer management. Based on logistic tumour growth law, the combination of chemotherapy with immunotherapy was recently simulated by Hu and colleagues [115] to address the antagonistic, additive and/or synergistic effect of the combined treatment *in silico*, in order to select an optimal chemotherapeutical protocol. The theoretical model simulates the dynamic evolution of tumour population density under dual coupling periodic interventions (treatment). The model outcome is in fine agreement with Loewe's additivity model, a universal reference model for drug interaction, showing that the curative effect of the combined treatment is strongly dependent on the intensity, nature (sub/supra-additive) and the timing of the individual treatments. This study could serve as a useful preclinical pharmacokinetic assessment of drug-drug or drug-immunotherapy interaction.

## 4. Conclusions

To further increase the power of complex tumour modelling into the future, greater depth of knowledge of tumour biology on an individual tumour basis will be required. This data is likely to be at micro(cellular) level as well as genetic and chemical level within the tumour. The data required for model input, necessitating the need for *in vivo* tumour measurement and/or imaging (before and at regular intervals during treatment), may include levels of specific protein synthesis and activity, a full description of tumour oxygenation from 0 to approximately 40 mm Hg, intrinsic radiosensitivity levels and cell kinetic behaviour of all cell types involved including all primary tumour cell types, vessel (endothelial cells), surrounding normal cell information, patient condition variable such as immune system responses in the form of DNA damage repair efficiency.

Specific difficulties of data gathering for tumours models are listed in Table 3 and generally include the following:

- variations between patients;
- the dynamic nature of the parameters desired;
- time/expense due to complexity of testing procedures, for example, protein analysis;
- understanding the mechanisms of microscopic processes within the tumour which are often interrelated.

While overcoming the difficulties imposed by these parameters can be a complicated task due to clinical confines in obtaining quantifiable data, there is always an option to undertake computational sensitivity studies of specific parameters within biologically plausible limits. 

The future of *in silico *tumour modelling is challenging, but wide open for expansion through the dedication of radiobiology, medical physics, and computational science researchers. Assumptions, estimations, idealisations, and trial-end-error situations will constantly be part of the modelling process. Nevertheless, despite its limitations, mathematical and computational modelling is already playing an integral role in several aspects of cancer management and it is expected to gain more ground in the near future by complementing other preclinical studies. 

## Figures and Tables

**Table 1:** Models that simulate tumour growth and/or radiotherapy, without tumour oxygenation considerations (avascular tumours).

Model details	Objectives	Key parameters	Model outcomes
Stochastic, *CELLSIM, and CELLGROW,* Donaghey, 1981–1983 [1, 2]	Individual cell/cell group growth model	Phase transition probabilities, proliferation-based commands on up to 90 cell groups, contact inhibition modelled	Monolayer cell growth achieved and RT/drug therapy applied

Mathematical, O'Donoghue, 1997 [3]	Modelling exponential proliferation at small tumour sizes and Gompertzian at larger sizes	3 LQ based parameters, 2 growth related parameters and 1 radiation induced death rate parameter	The effects of RT described in terms of cure (clonogenic cell sterilization) and/or tumour regression/regrowth

Stochastic, Marcu et al., 2002–2006 [4, 5]	Modelling tumour growth and response to radiotherapy	Cell cycle specific surviving fractions (SF_2_) based on LQ model. Repopulation mechanisms during RT such as cell recruitment, accelerated stem cell division, and asymmetry loss in stem division	The effect of conventional and altered fractionation radiotherapy on cell survival evaluated. The contribution and likeliness of repopulation mechanisms assessed

**Table 2:** Models that simulate tumour growth and/or radiotherapy, incorporating tumour oxygenation (vascularised tumours).

Model details	Objectives	Key parameters	Model outcomes
Mathematical, Tannock, 1972 [6]	To relate oxygen tension, radiosensitivity, and distance from blood vessels	Distance to blood vessel (radial), pO_2_ vessel radius, coefficient of diffusion, rate of O_2_ consumption, diffusion maximum radius	A full range of oxygen tension values are required to accurately model tissue oxygenation and radiosensitivity with good agreement to clinical data

Stochastic, Duchting et al., 1981–1995 [7–10]	To grow and treat *in vitro* tumour spheroids (mouse brain and lung) in a nutrient medium as well as the surrounding normal tissue (epidermis)	Rapid versus medium or slow proliferation (CCT adjustment 10 to 30 hours), 120 hour dead cell clearance, LQ radiosensitivity with separate *α*_hypoxic_ and *β*_hypoxic_ terms, 30% repair probability of sublethal damage, 10% G_0_ phase recruitment, individual cell cycle phase times	Six RT schedules simulated and compared in terms of cell kill to assess TCP and likelihood of epidermal side effects, 3 no./day produced high toxicity and a reduction from 60 to 50 Gy total doses is suggested

Stochastic, Kocher et al., 1997–2000 [11, 12]	Brain tumour growth and RT in a regular 3D lattice	Cell cycle times of 2 or 5 days, regular capillary placement in the lattice, 100 *μ*m oxygen diffusion limit to define hypoxia and 140 *μ*m to define necrosis, constant OER value of 3.0, 5 day dead cell clearance	Three RT schedules simulated, with accelerated RT more effective on fast growing tumours

Mathematical, Wouters and Brown, 1997 [13]	Equation-based modelling of hypoxic tumour LQ cell kill for tumours with a 2-compartment oxygen level make-up versus intermediate (0.5 to 20 mm Hg) oxygen values	Radial distance of a cell from the tumour boundary to determine oxygenation (2-component model or complete range of pO_2_ values considered)	Small impact of full reoxygenation between fractions: hypoxia plays a significant role in determining outcome, 10% hypoxia and 30 × 2 Gy radiotherapy equates to 10^4^ times less cell kill using a full pO_2_ range compared to the 2-component oxygenation model

Stochastic, Stamatakos et al., 2001–2010 [14–17]	Simulating lung and brain tumour growth in a 3D lattice to determine optimal individualised RT schedules	Gaussian probability cell cycle times, G_0_ phase on 25 hours, reoxygenation during shrinkage, S phase versus non-S phase LQ radiosensitivity values, cellular hypoxia if more than three cells from nutrient source, OER ranging from 1.0 to 3.0 with separation into OER_*α*_ and OER_*β*_	OER_*β*_ values of 3.0 to 3.5 provide cell kill in agreement with cell culture survival curves, accelerated schedules are beneficial, wild type tumours (higher *α*/*β*) respond well compared to mutated tumours

Mathematical, Nilsson et al., 2002 [18]	Simulating realistic oxygenation gradients and cell densities to explore their impact on radiosensitivity at both the microscopic and macroscopic scale	Oxygenation, vessel geometry parameters (density, radius, heterogeneity), oxygen consumption rate, distance from a vessel	Vascular heterogeneity impacts significantly on the hypoxic fraction, local and global dose responses are predicted from LQ theory using the initial clonogenic cell number and the effective radiation distance

Mathematical (stochastic components), Popple et al., 2002 [19]	Predicting tumour control probability after selective boosting hypoxic subvolumes within a tumour mass	Reoxygenation between doses, OER of 2.0 for hypoxic cells, boost and nonboost spatial cell compartments.	A 20% to 50% boost in dose to a subpopulation of hypoxic cells increased tumour control probability equal to that of an oxic tumour, a boost dose to regions of transient hypoxia has little effect

Stochastic, Borkenstein et al., 2004–2010 [20–22]	Simulating hypoxic tumour growth and RT considering hypoxia and angiogenesis	OER = 2.5 and 3.0 (continuous cell oxygenation range in later work), vessels modelled in a regular lattice, angiogenetic factors to induce vessel growth and hence pO_2_ delivery to cells, distance of a cell from a vessel.	An increase in capillary cell cycle time affects tumour doubling time as does the intercapillary distance, doses of 86 Gy versus 78 Gy are required to control the simulated tumours for conventional and accelerated schedules, respectively

Mathematical (stochastic components), Daşu et al., 1999–2009 [23–26]	Simulating 2D cell distributions to investigate the effects of cell heterogeneity, hypoxia (acute and chronic) on RT outcome	2-compartment oxygenation (2.5 mm Hg hypoxic threshold) versus full oxygenation range, cell heterogeneity	Temporal oxygenation changes between treatment fractions are less important than the presence of chronic hypoxia, and a small degree of hypoxia during every treatment fraction has an effect on tumour response regardless of the changes in spatial hypoxia, a 2-component hypoxia model is not sufficient in describing tumour oxygenation

Mathematical (stochastic components), Søvik et al., 2007 [27]	Optimising tumour control through redistribution of the delivered dose, “dose painting”	pO_2_ histograms (0 to 102.5 mm Hg), hypoxia defined by 5.0 mm Hg threshold, reoxygenation modelled, heterogeneous cell density, dose delivery based on four pO_2_ thresholds: 2.5, 5.0, 20.0, 102.5 mm Hg, OER_*α*,max⁡_ and OER_*β*,max⁡_ of 2.5 and 3.0, OER equation maximum of 3.28	Prescribing varying doses to different parts of the tumour can significantly increase TCP although the rate of reoxygenation is crucial. Tumours with no reoxygenation have the most benefit of dose redistribution. Chronic hypoxia influences outcome more than acute hypoxia

Stochastic, Titz and Jeraj, 2007 [28]	Simulating cell line specific parameters and functional pre-treatment 3D PET/CT data to investigate the effects of oxygenation on RT outcome	5-day cell dead clearance, 36-hour average cell cycle time, OER with *K* value of 3.0, full pO_2_ range, 1 mm Hg necrotic threshold, individual phase radiosensitivities	Tissue growth curves and reoxygenation data follow *in vitro* and human clinical data, with an accurate time delay of tumour shrinkage predicted

Stochastic, “*HYP-RT,*” Harriss-Phillips et al., and Tuckwell et al., 2008–2011 [29, 30]	Simulating hypoxic tumour growth and reoxygenation during RT of HNSCC	LQ-based cell kill with OER consideration, full cellular pO_2_ distribution (1 to 100 mm Hg), OER curve changing with dose per fraction, reoxygenation as well as accelerated repopulation between dose fractions	Hyperfractionation using 2 × 1.1 Gy per day is optimal for HNSCC, hypoxic tumours require 16 Gy extra dose during conventional radiotherapy compared to oxic tumours, and the maximum value and shape of the oxygen enhancement ratio curve that may be dependent on dose per fraction are crucial for prediction of TCP

**Table 3:** Difficulties in tumour growth and treatment response modelling relating to suitable input biological data.

Parameters/information that are difficult to obtain (quantitatively)	Reason for the difficulty	Overcoming the difficulty
Aggressiveness and time of onset of accelerated repopulation	Intertumour variability that is unknown cannot measure/estimate for individual patients without cell biopsy sample used in *in vitro* tests which may alter results	Grouping patients into tumours that are likely to have slow or fast repopulation by some means of genetic/pathologic testing—however methods currently unknown

The extent of various mechanisms responsible for tumour repopulation during treatment	The interplay between recruitment, accelerated stem division, abortive division and loss of asymmetrical division, in stem cells makes it difficult to evaluate their individual effect	Research stem cell properties for rapidly proliferating tumours.Sensitivity study on each individual and combined parameter when modelling

Input of individualised tumour data, for example, intrinsic radiosensitivity, differences in stem/transit or quiescent cell radiosensitivity	Currently no pretreatment testing due to logistics and time of testing	Research cell type/proliferative capacity-dependent radiosensitivities for different tumour cell lines, individualised radiosensitivity pre-treatment testing (requires staff/money/time)

Tumour oxygenation/reoxygenation	Different in every tumour, changes in time, access to equipment, for example, daily/weekly PET, invasive nature of *in vivo* quantitative data gathering, for example, Eppendorf/Oxy Lab probe	Access and research into to the feasibility and drug development for daily/weekly PET scans, with tracers that can image hypoxic regions with various thresholds, for example, 2.5, 5.0, 10 mm Hg

Drug pharmacokinetics	Lack of quantitative *in vivo* assays	Using *in vitro* data if existent, parameter estimation and sensitivity study. Molecular pharmacological modelling is required

Cell survival data for chemotherapy	Lack of mathematical formalism equivalent to the LQ model used in radiotherapy	Using *in vitro* data if existent for that particular agent
